# Development and validation of a radiomics-based model to predict local progression-free survival after chemo-radiotherapy in patients with esophageal squamous cell cancer

**DOI:** 10.1186/s13014-021-01925-z

**Published:** 2021-10-12

**Authors:** He-San Luo, Ying-Ying Chen, Wei-Zhen Huang, Sheng-Xi Wu, Shao-Fu Huang, Hong-Yao Xu, Ren-Liang Xue, Ze-Sen Du, Xu-Yuan Li, Lian-Xin Lin, He-Cheng Huang

**Affiliations:** 1 Department of Radiation Oncology, Shantou Central Hospital, Shantou, 515000 Guangdong China; 2 Department of Medical Oncology, Huizhou Municipal Central Hospital of Guangdong Province, Huizhou, China; 3 Department of Surgical Oncology, Shantou Central Hospital, Shantou, Guangdong China; 4 Department of Medical Oncology, Shantou Central Hospital, Shantou, Guangdong China

**Keywords:** Chemo-radiotherapy, Esophageal squamous cell cancer, Radiomics, LPFS, Nomogram

## Abstract

**Purpose:**

To develop a nomogram model for predicting local progress-free survival (LPFS) in esophageal squamous cell carcinoma (ESCC) patients treated with concurrent chemo-radiotherapy (CCRT).

**Methods:**

We collected the clinical data of ESCC patients treated with CCRT in our hospital. Eligible patients were randomly divided into training cohort and validation cohort. The least absolute shrinkage and selection operator (LASSO) with COX regression was performed to select optimal radiomic features to calculate Rad-score for predicting LPFS in the training cohort. The univariate and multivariate analyses were performed to identify the predictive clinical factors for developing a nomogram model. The C-index was used to assess the performance of the predictive model and calibration curve was used to evaluate the accuracy.

**Results:**

A total of 221 ESCC patients were included in our study, with 155 patients in training cohort and 66 patients in validation cohort. Seventeen radiomic features were selected by LASSO COX regression analysis to calculate Rad-score for predicting LPFS. The patients with a Rad-score ≥ 0.1411 had high risk of local recurrence, and those with a Rad-score < 0.1411 had low risk of local recurrence. Multivariate analysis showed that N stage, CR status and Rad-score were independent predictive factors for LPFS. A nomogram model was built based on the result of multivariate analysis. The C-index of the nomogram was 0.745 (95% CI 0.7700–0.790) in training cohort and 0.723(95% CI 0.654–0.791) in validation cohort. The 3-year LPFS rate predicted by the nomogram model was highly consistent with the actual 3-year LPFS rate both in the training cohort and the validation cohort.

**Conclusion:**

We developed and validated a prediction model based on radiomic features and clinical factors, which can be used to predict LPFS of patients after CCRT. This model is conducive to identifying the patients with ESCC benefited more from CCRT.

**Supplementary Information:**

The online version contains supplementary material available at 10.1186/s13014-021-01925-z.

## Introduction

Esophageal cancer (EC) is the sixth common malignant tumors in China with an estimated 477.9 thousand new cases, accounting for half of the new esophageal cancer worldwide [1, 2]. In China, approximately 90% of the patients with esophageal cancer are histologically diagnosed as esophageal squamous cell carcinomas (ESCC) which is different from esophageal adenocarcinoma (EAC) in risk factors and prognosis [3]. Most patients with locally advanced ESCC lost the opportunity for surgery, and concurrent chemo-radiotherapy (CCRT) has been recommended as a standard treatment [4]. However, more than half of patients treated with standard dose CCRT eventually developed local recurrence or distant metastases and succumbed to this disease [5, 6]. A individual CCRT strategy with escalated radiation dose based on PET-CT would benefit the patients with high tumor burden and risk of recurrence [7, 8]. To facilitate a individual CCRT strategy in an early stage, solid predictive model for local recurrence and prognosis could play an important role.

For patients received CCRT, local and regional recurrence is the most common failure pattern and pre-treatment clinical TNM staging is still the most commonly used system for prognosis prediction [9]. However, the currently used clinical TNM staging follows the same criteria as pathological staging, which is based on imaging assessment of tumor size and surrounding invasion, ignoring the information such as length and volume of esophageal cancer lesions. Recently, a series of clinicopathologic biomarkers have been investigated and verified to be available for prediction of therapeutic response and prognosis [10–12]. Radiomics is a new technique for image quantitative analysis about computed tomography (CT) images, magnetic resonance (MR) images, positron emission tomography (PET) images, etc. [13]. Several studies demonstrated that radiomic features could potentially identify prognostic phenotype in patients with EC. Yip et al. [14] suggested that a model combined CT-based texture feature and esophageal maximal wall thickness assessment could predict the overall survival in EC patients treated with CCRT. Moreover, the model performed better than treatment response alone. Larue et al. [15] extracted out five radiomic features from CT image before chemoradiotherapy to describe the heterogeneity of tumors and found that these five features could predict the 3-year survival rate of patients with EC after neo-chemoradiotherapy. However, most radiomic studies included a small number of patients with EAC and ESCC.

In this study, we explored the prognostic value of 3D radiomic features from pretreatment CT images of esophageal cancer patients and developed a model combined radiomic features and clinical information to predict LPFS in patients with ESCC after CCRT. To evaluate the performance of the model, a validation cohort of patients were employed for validation.

## Patients and methods

### Patients’ cohort

We collected the clinical data of patients diagnosed as ESCC and received CCRT in our hospital during the period from January 2013 to December 2015. Patients were excluded if they met the exclusion criteria as follows: (1) patients received esophagectomy and preoperative or postoperative adjuvant radiotherapy; (2) patients had distant metastatic disease; (3) patients received low-dose (< 50 Gy) palliative radiotherapy; (4) clinicopathological information of the patients was incomplete; (5) patients were diagnosed as esophageal fistula before treatment; (6) poor visualization quality due to image artifacts or the tumor was too small to be recognized on CT images; (7) patients had other primary tumor; (8) patients died within three months after chemoradiotherapy.

**Fig. 1:**
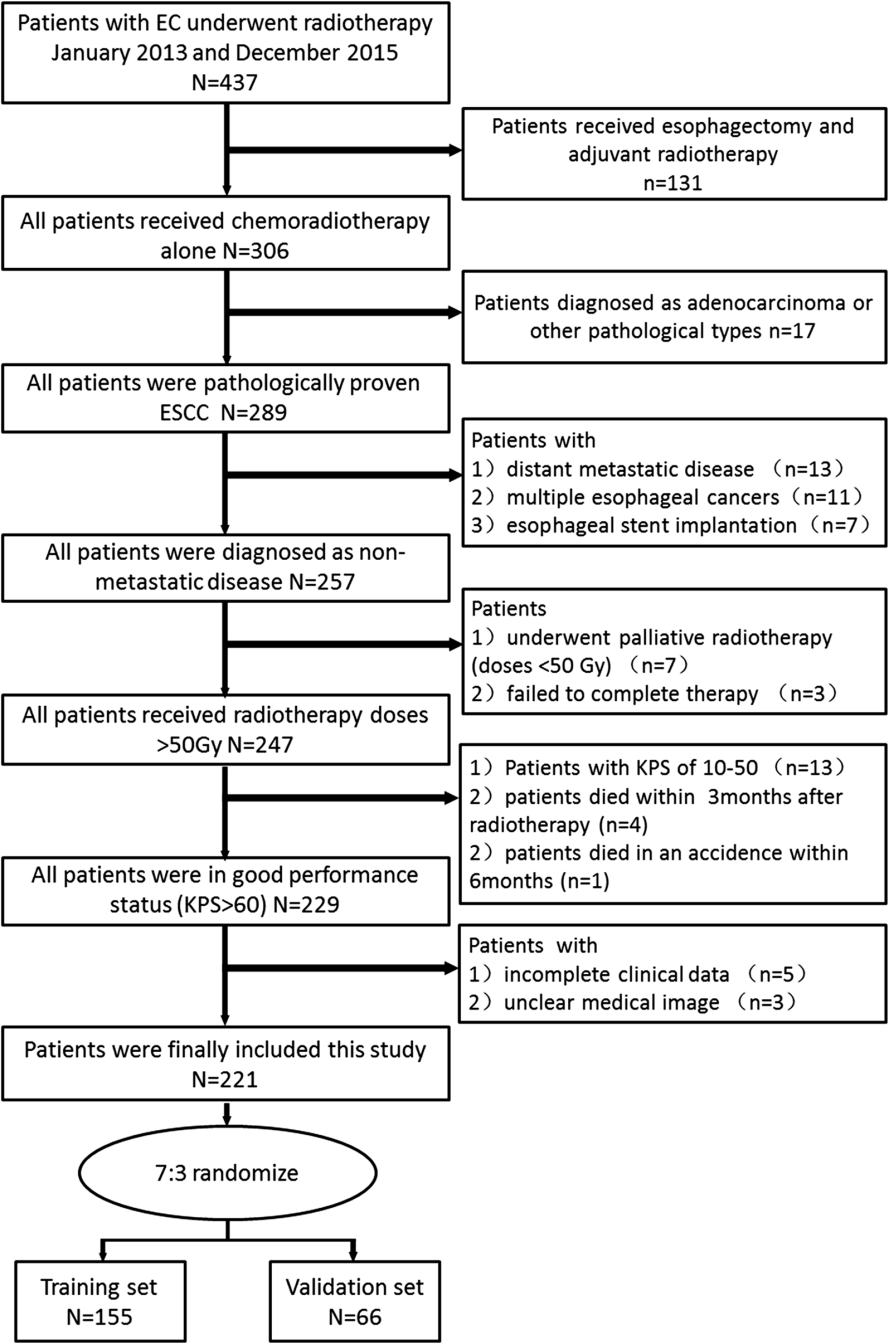
Flow chart of patients’ screening and allocation

After multiple iterations, a total of 221 patients were randomly divided into two groups, with 155 patients in the training cohort and 66 patients in the validation cohort. To improve the generalization property of the result, multi-factors stratification was used to keep the characteristics of sub cohort consistent with the whole cohort. The process of patients’ enrollment and randomization were shown in Fig. 1. This study was approved by the Institutional Committee of our hospital on Human Rights. Disease of the patients was staged according to the 8th edition of AJCC TNM classification for esophageal cancer [16].

### Chemoradiotherapy protocol

Radiotherapy was delivered daily to patients with three-dimensional conformal radiation therapy (3DRT) or intensity-modulated radiation therapy (IMRT) technique using a Varian IX or Varian 23EX linear accelerator in this study. The gross tumor volume (GTV) includes the esophageal cancer (GTVp) and the positive regional lymph nodes (GTVnd). The GTV was delineated on CT imaging according to barium esophagogram, endoscopic examination or PET imaging. The CTV was defined as the GTVp with 0.5–1 cm radial expansion and 2.5–3 cm axial direction expansion or the GTVnd with 0.5–0.8 cm uniform expansion. The planning target volume (PTV) was defined as CTV with a 1 cm uniform expansion. A total prescribed dose of 50–72 Gy (median, 64 Gy) in conventional fractionation was delivered to the patients.

Two cycles of platinum-based chemotherapy were administered concurrently with radiotherapy. Sixty-one patients received TP (paclitaxel + cisplatin) chemotherapy every three weeks, which consists of cisplatin (60 mg/m^2^ on Day 1) plus paclitaxel (135–180 mg/m^2^ on Days 1). One hundred and sixty patients received the PF (cisplatin + fluorouracil) regimen every four weeks, which consists of cisplatin (60 mg/m^2^ on Day 1) and fluorouracil (750 mg/m^2^ /24 h on Days 1–4).

### Response evaluation

The response to chemo-radiotherapy was evaluated one month after CCRT according to the criteria of short-term response evaluation standard on esophageal cancer using CT images and barium esophagogram. According to the response evaluation criteria, clinical response was classified as complete response (CR), partial response (PR), no response (NR), or progressive disease (PD). Patients who were classified as CR by barium esophagogram and had the maximal esophageal wall thickness of ≤ 1.2 cm and the volumes of residual lymph nodes of ≤ 1.0 cm^3^ on CT were finally defined as CR [17].

### Radiomic feature extraction

All patients were scanned using GE Lightspeed 64-slice spiral CT (GE Medical systems, Milwaukee, Wis) before radiotherapy. CT image acquisition was performed according to the following acquisition protocol: The CT tube voltage was 120 kV and the tube current was 120 mAs. Rack rotation time: 0.6 s; Detector collimation parameters: 64 × 0.625 mm; field of view (FOV): 400-500 mm; Matrix: 512 × 512; Layer thickness is 5 mm, layer spacing is 5 mm. Contrast medium was injected with a high-pressure syringe at a flow rate of 3.0 ml/s (1–1.5 ml/kg, ioproxamine injection 300), followed by 30 to 40 ml of normal saline for flushing, and late arterial CT images were collected with a delay of 30 s. To reduce the variability between images from different patients, all images were resampled to voxel of 1*1*1mm^3^.

3D Slicer (version, 4.10.2, Stable Release) with radiomics extension was used for image segmentation to obtain volume of interest (VOIs). The primary tumor volume (GTV) delineated by radiation oncologists for radiotherapy treatment planning design was defined as VOI for radiomic features extraction. Any pixel with an attenuation of less than − 50 HU was excluded to avoid adjacent air, fat, blood vessels and surrounding organs. Image segmentation was performed independently by a radiation oncologist and another radiologist. To assess the reproducibility of the radiomic features extraction, tumor segmentation was performed again two months later by the same radiologist in 30 randomly chosen patients.

Pyradiomics V3.6.2 was used to extract radiomic features from delineated VOIs. Several categories of features were extracted from VOIs, including first order statistics features (IH, intensity histogram), shape-based histogram features, and texture features (gray-level co-occurrence matrix, GLCM; gray-level size-zone matrix, GLSZM; gray-level run-length matrix, GLRLM; neighboring gray-tone difference matrix, NGTDM; and gray-level dependence matrix, GLDM). The wavelet filter was used in image pre-processing for texture features extraction. In all, for each VOI, 107 original features (Additional file 1: Table S1) and 744 wavelet features (Additional file 1: Table S1) were collected. Among the 107 original features, there were 18 first order statistics features, 14 shape-based histogram features, 24 GLCM features, 14 GLDM features, 16 GLRLM features、16 GLSZM features and 5 NGTDM features. Mathematical definitions of these radiomic features have previously been described [18] and available at https://pyradiomics.readthedocs.io/en/latest/features.html.

### Statistical analysis

At the first, statistical analyses were performed with Chi-squared test or Fisher’s to assess the difference of the clinical characteristics between training cohort and validation cohort. A *p*-value of < 0.05 was considered statistically significant.

In the pre-processing of radiomic features, all the values of radiomic features were normalized using Z-score normalization, which made features values lying within similar ranges and reduced the influence of large discrete values. The intra-class correlation coefficient (ICC) analysis was performed to evaluate the reproducibility of each radiomic feature. Only the features with ICCs values ≥ 0.900 were selected for further analysis. Then, the least absolute shrinkage and selection operator (LASSO) with COX regression was performed using R software version 3.6.2 (R Foundation for Statistical Computing, Vienna, Austria) to identify the features associated with LPFS in the training cohort. The optimal parameter lambda (λ) was chosen from the LASSO model using tenfold cross-validation with the minimum partial likelihood deviance. Radiomic feature score (Rad score) for each patient was built based on the LASSO COX regression model in the training cohort. The LASSO COX regression formula:

$${\text{Rad}}\,{\text{score}} =\upbeta 1{\text{X}}1 +\upbeta 2{\text{X}}2 +\upbeta 3{\text{X}}3 + \cdots +\upbeta {\text{nXn}}$$

In the above formula, X1, X2 … Xn are the different radiomic features identified by the LASSO COX regression model, and β1, β2 … βn are the regression coefficients of the corresponding features in the regression model.

Univariate analysis was performed to identify the potential prognostic factors associated with LPFS. Multivariable COX regression analysis was performed to identify the independently predictors for LPFS. A nomogram model combined Rad-score and clinical factors for predicting LPFS was developed and validated based on the results of multivariable COX regression analysis using rms package and foreign package in R software. The predictive accuracy of the nomogram model was assessed using Calibration curve validation in both training cohort and validation cohort. All the analyses were performed with R software version 3.6.2.

## Results

### Patients’ characteristics

**Fig. 2:**
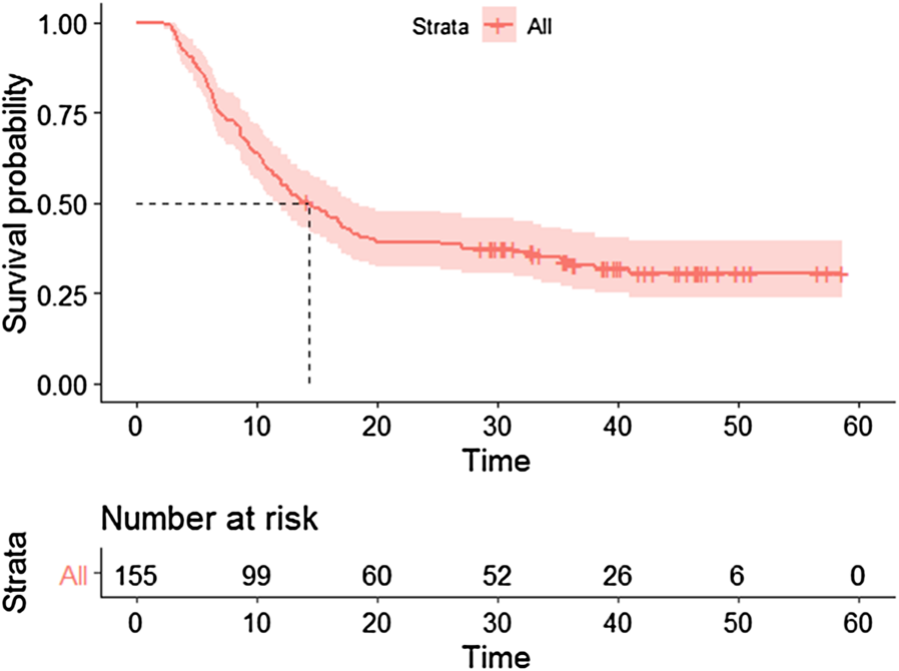
Kaplan–Meier curve of local-progression free survival for all patients

A total of 221 ESCC patients who received chemoradiotherapy in our hospital were eligible for further analysis in this study. Patients’ characteristics were summarized in Table 1. The median follow-up time was 18.6 months. By the end of the last follow-up, 153 patients developed local regional disease progression or died. The median LPFS in the whole group was 13.7 months, and the rates of 1-year, 2-year and 3-year LPFS were 56.1%, 37.4% and 32.1%, respectively (Fig. 2).

**Table 1:** Comparison of patients’ characteristics between training cohort and validation cohort

Variables	Training cohort (n = 155)	Validation cohort (n = 66)	χ^2^/*t*	*p*
Age (years), Mean ± SD	65.7147 ± 9.74	64.73 ± 10.16	0.678	0.499
Gender			0.342	0.559
Male	116 (74.8)	45 (68.2)		
Female	39 (25.2)	21 (31.8)		
Tumor location			5.814	0.121
Cervical	6 (3.9)	8 (12.1)		
Upper thoracic	34 (21.9)	16 (24.2)		
Middle thoracic	91 (58.7)	33 (50.00)		
Lower thoracic	24 (15.5)	9 (13.6)		
T stage			3.193	0.363
T1	2 (1.3)	0 (0)		
T2	11 (7.1)	9 (13.6)		
T3	66 (42.6)	27 (40.9)		
T4	76 (49.0)	30 (45.5)		
N stage			1.856	0.603
N0	20 (12.9)	13 (19.7)		
N1	70 (45.2)	28 (42.4)		
N2	55 (35.5)	22 (33.3)		
N3	10 (6.5)	3 (4.5)		
Clinical stage			3.152	0.369
I	2 (1.3)	0 (0)		
II	15 (9.7)	11 (16.7)		
III	88 (56.8)	37 (56.1)		
Iva	50 (32.3)	18 (27.3)		
Radiation dose, Median (range)	64 (60–66)	64 (60–66)	− 0.920	0.358
LDH group			1.282	0.258
High	88 (56.8)	32 (48.5)		
Normal	67 (43.2)	34 (51.5)		
NLR, Median (range)	2.73 (1.96–3.71)	2.76 (2.00–3.63)	− 0.448	0.654
PLR, Median (range)	137.78 (100.56–181.43)	138.87 (101.31–182.26)	− 0.344	0.731
CR ratio	52 (33.5)	26 (39.4)	0.693	0.405
Rad-score, Mean ± SD	− 0.0289 ± 0.35	− 0.058 ± 0.538	0.474	0.636

In order to develop and validate a radiomics-based model for predicting LPFS of the patients, they were randomly divided into training cohort and validation cohort. There were 155 patients in the training cohort and 66 patients in the validation cohort. No significant differences (All *p* > 0.05) were found between the distribution of baseline characteristics in two cohorts, such as age, gender, tumor location, T stage, N stage, clinical staging, lactate dehydrogenase (LDH), neutrophil to 1ymphocyte ratio (NLR), platelet to lymphocyte ratio (PLR) and CR ratio (33.5% in the training cohort vs 39.4% in the validation cohort). Therefore, the two cohorts of patients were comparable.

### Rad-score building based on radiomic features

**Fig. 3:**
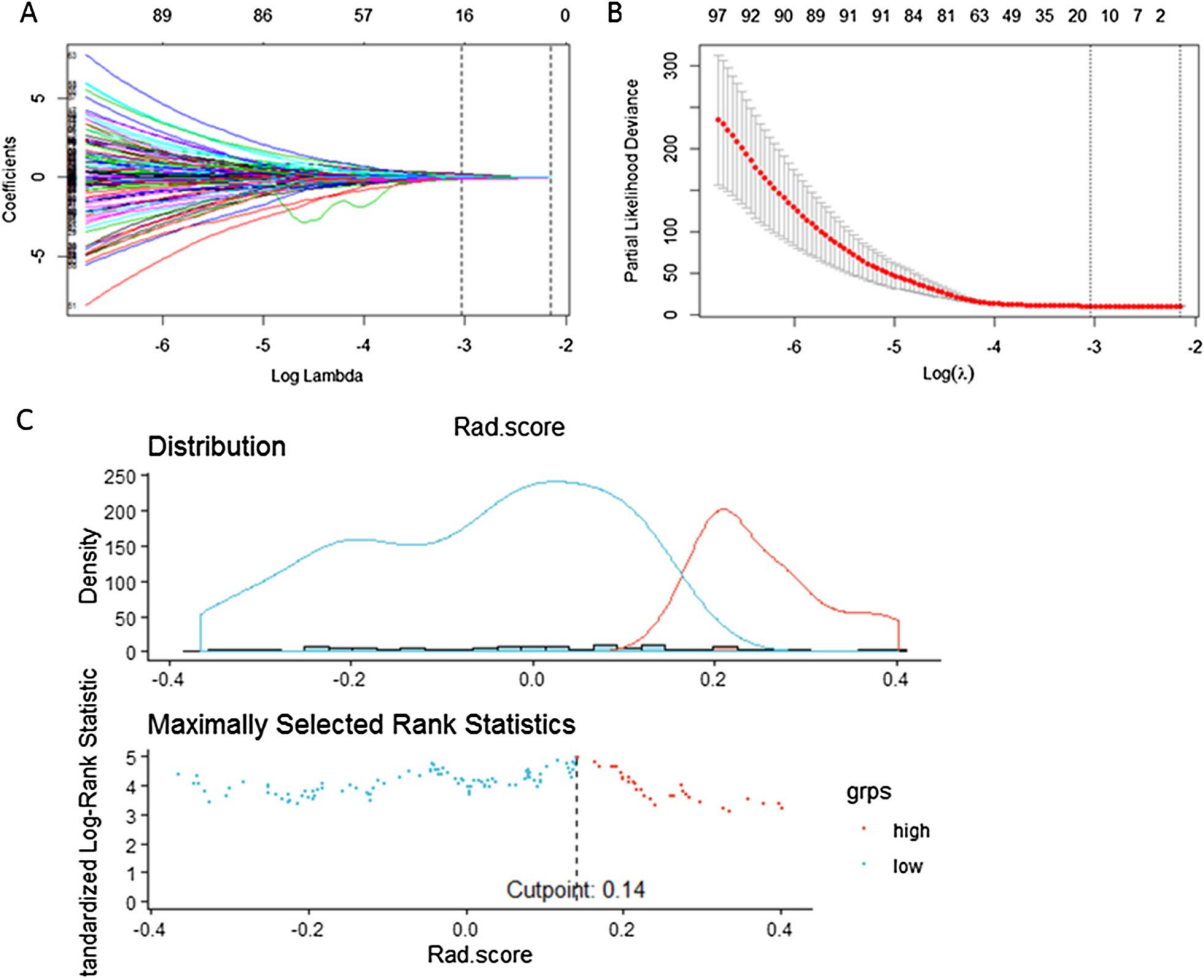
Selection of radiomic features associated with LPFS using the LASSO COX regression model. **A** Coefficients profiles of radiomic features. The horizontal axis value is logλ, and the vertical axis value represent the coefficients of radiomic features. **B** The cross-validation curve. The horizontal axis value is logλ, and the vertical axis value is partial likelihood deviance. **C** The optimal cutoff of Rad-score. Red lines or red dots represent patients at high risk of local recurrence and green lines or green dots represent patients at low risk of local recurrence. The optimal cutoff value is 0.1411, as shown in the vertical line in the figure

LASSO-COX regression was used to screen out the optimal radiomic features associated with LPFS of the patients in the training cohort (Fig. 3A, B). As a result, seventeen radiomic features were screened out (The features and their coefficients were listed in the Table 2). The Rad-score was calculated as follows: Rad-score = -0.104667846*original_firstorder_Skewness + 0.001161134*origin_glszm_SizeZoneNonUniformityNormalized + 0.034339901*origin_glszm_SizeZoneNonUniformity-0.017089976*origin_glszm_LowGrayLevelZoneEmphasis + 0.062595767*wavelet-HLL_glcm_Idn + 0.026703955*wavelet-HLL_firstorder_Maximum + 0.042957143*wavelet-HLL_glszm_SizeZoneNonUniformityNormalized + 0.017543973*wavelet-LHL_firstorder_TotalEnergy + 0.003781538*wavelet-LHL_firstorder_Maximum-0.007364328*wavelet-LLH_gldm_SmallDependenceLowGrayLevelEmphasis + 0.157807433*wavelet-LLH_glcm_DifferenceVariance + 0.042028490*wavelet-LLH_glrlm_ShortRunHighGrayLevelEmphasis-0.101981005*wavelet-LLH_ngtdm_Coarseness-0.073958943*wavelet-HLH_gldm_SmallDependenceLowGrayLevelEmphasis + 0.051287394*wavelet-HLH_firstorder_Maximum-0.055239045*wavelet-HHH_glcm_MaximumProbability-0.028958889*wavelet-LLL_glcm_Imc2.

**Table 2:** Radiomics feature associated with LPFS selected by LASSO COX analysis

Radiomics features	Coefficients
original_firstorder_Skewness	− 0.104667846
origin_glszm_SizeZoneNonUniformityNormalized	0.001161134
origin_glszm_SizeZoneNonUniformity	0.034339901
origin_glszm_LowGrayLevelZoneEmphasis	− 0.017089976
wavelet-HLL_glcm_Idn	0.062595767
wavelet-HLL_firstorder_Maximum	0.026703955
wavelet-HLL_glszm_SizeZoneNonUniformityNormalized	0.042957143
wavelet-LHL_firstorder_TotalEnergy	0.017543973
wavelet-LHL_firstorder_Maximum	0.003781538
wavelet-LLH_gldm_SmallDependenceLowGrayLevelEmphasis	− 0.007364328
wavelet-LLH_glcm_DifferenceVariance	0.157807433
wavelet-LLH_glrlm_ShortRunHighGrayLevelEmphasis	0.042028490
wavelet-LLH_ngtdm_Coarseness	− 0.101981005
wavelet-HLH_gldm_SmallDependenceLowGrayLevelEmphasis	− 0.073958943
wavelet-HLH_firstorder_Maximum	0.051287394
wavelet-HHH_glcm MaximumProbability	− 0.055239045
wavelet-LLL_glcm_Imc2	− 0.028958889

**Fig. 4:**
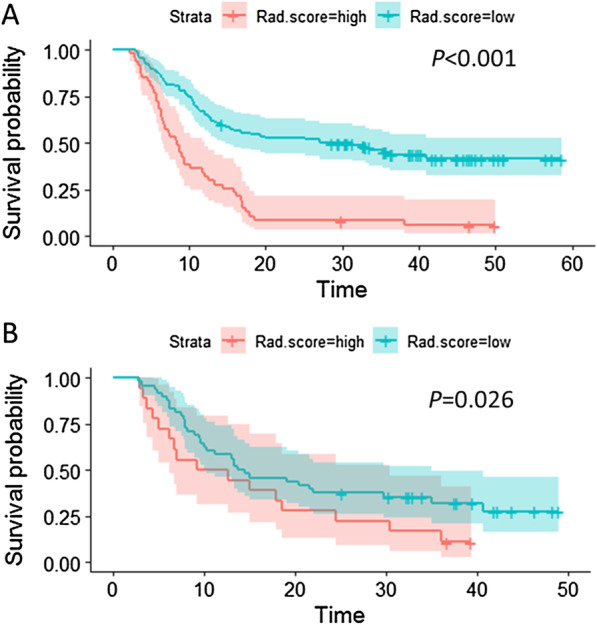
Kaplan–Meier survival curve of patients with high and low recurrence risk based on Rad-score. **A** LPFS survival curve of patients in the training cohort: green line represents patients with low risk of local recurrence and red represents patients with high risk of local recurrence. The difference is significant between two groups, *p* < 0.001. **B** LPFS survival curve of patients in the validation cohort: green line represents patients with low risk of local recurrence and red line represents patients with high risk of local recurrence. The difference is significant between two groups, *p* = 0.026

There was an optimal cutoff value of Rad score to divide the patients into two groups with different risk of local recurrence. As shown in Fig. 3C, the patients with a Rad-score ≥ 0.1411 had high risk of local recurrence, and those with a Rad-score < 0.1411 had low risk of local recurrence. In the training cohort, the patients in the group with high risk of local recurrence had significantly shorter time of LPFS than those with risk of local recurrence (Fig. 4A, HR 2.882, 95% CI 1.926–4.313, *p* < 0.001). The same result was found in the validation cohort (Fig. 4B, HR 1.997, 95% CI 1.070–3.728, *p* = 0.026).

### Development and validation of a predictive nomogram based on Rad-score

**Fig. 5:**
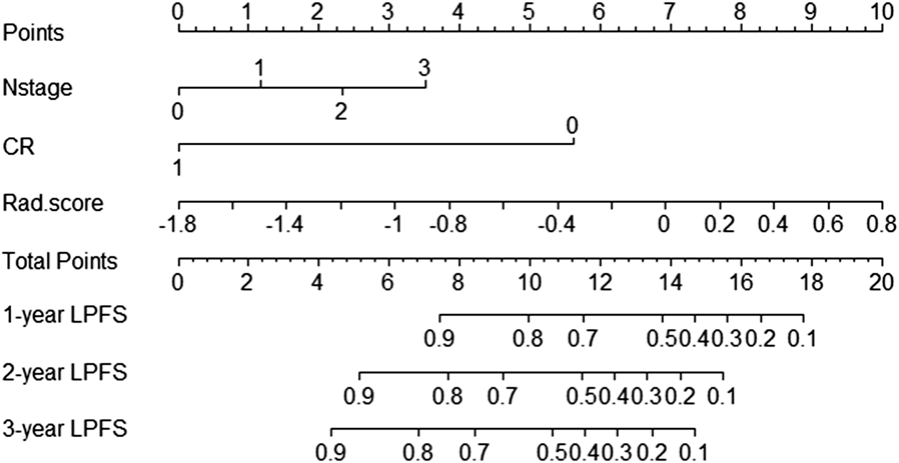
Nomogram model for predicting LPFS based on Rad-score. Rad.score refers to Rad-score. 0, 1, 2, and 3 refers to N0, N1, N2 and N3 in N stage line respectively. CR represents complete response, the value of 0 and 1 refer to non-CR and CR status respectively

In order to develop a model to predict LRFS based on multiple factors, we performed univariate and multivariate analyses to identify predictive factors for LPFS. Univariate analysis showed that the T stage, N stage, clinical stage and CR status were significantly associated with LPFS both in training cohort and validation cohort (Table 3). Multivariate analysis showed that N stage, CR status and Rad-score were independent predictive factors for LPFS in ESCC patients after chemoradiotherapy (Table 4). A nomogram model for predicting LPFS was built based on the result of multivariate analysis (Fig. 5A). As shown in Fig. 5 1-year, 2-year and 3-year LPFS probability of every patient could be predicted based on the independent clinical characteristics and Rad-score. The C-index of the nomogram was 0.745 (95% CI 0.7700–0.790) in training cohort and 0.723(95% CI 0.654–0.791) in validation cohort.

**Table 3:** Univariate analysis of prognostic factors associated with LPFS in patients with ESCC treated with chemoradiotherapy

Variables	Training cohort *p*			Validation cohort *p*		
	HR	95% CI	*p*	HR	95% CI	*p*
Age	0.987	0.967–1.007	0.202	0.984	0.957–1.011	0.252
Gender	1.639	1.014–2.650	0.044	1.198	0.652–2.202	0.560
Tumor location	1.120	0.851–1.473	0.419	1.289	0.917–1.812	0.143
T stage	2.015	1.453–2.793	< 0.001	1.943	1.227–3.077	0.005
N stage	1.867	1.446–2.410	< 0.001	1.765	1.215–2.563	0.003
Clinical stage	2.194	1.581–3.044	< 0.001	2.309	1.440–3.704	0.001
Radiation dose	0.965	0.923–1.009	0.118	0.996	0.921–1.078	0.927
LDH	1.641	1.116–2.414	0.012	1.369	0.778–2.408	0.276
NLR	1.069	0.965–1.184	0.199	1.015	0.901–1.142	0.810
PLR	1.001	0.999–1.003	0.177	1.001	0.999–1.004	0.377
CR status	0.128	0.072–0.228	< 0.001	0.295	0.157–0.556	< 0.001

**Table 4:** Multivariate analysis of prognostic factors associated with LPFS for patients with ESCC treated with chemoradiotherapy

Variables	Multivariate analysis *p*		
	HR	95% CI	*p*
T stage	0.858	0.526–1.400	0.540
N stage	1.892	1.122–3.190	0.017
Clinical stage	0.627	0.313–1.258	0.189
Rad-score	4.423	1.993–9.814	0.000
CR status	0.154	0.080–0.297	0.000

**Fig. 6:**
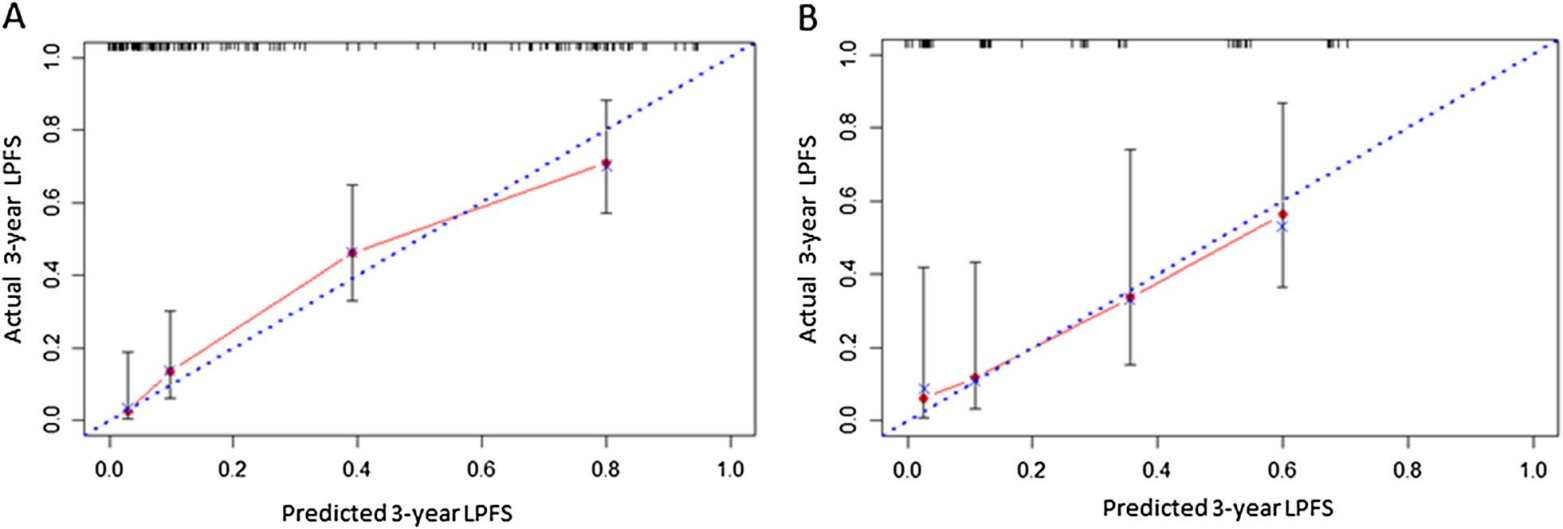
Calibration curve validation for Nomogram model in training cohort (**A**) and validation cohort (**B**). The horizontal axis represents the predicted 3-year LPFS and the vertical axis represents the actual 3-year LPFS. The blue diagonal dot line represents the ideal nomogram, and the red line represents the observed nomogram. The closer the calibration curve is to the diagonal line, the higher the consistency between the predicted results and the actual situation

Finally, we performed calibration curve to evaluate the accuracy of the nomogram model. As shown in Fig. 6, the 3-year LPFS rate predicted by the nomogram model based on Rad-score was highly consistent with the actual 3-year LPFS rate both in the training cohort and the validation cohort.

## Discussion

Concurrent chemoradiotherapy (CCRT) is a radical treatment for patients with inoperable esophageal cancer or refused surgery [4]. Many studies have shown that dose-escalation radiotherapy properly can improve the local control and survival of patients with ESCC [19–22]. Nevertheless, 30–50% of patients have local recurrence within 3 years [23–25]. In our present study, we constructed a prediction model combined the clinical characteristics and CT radiomic features which can predict the LPFS of patients after CCRT. With the help of this model, we can preliminarily judge the probability of LPFS of patients and identify the patients benefit more from CCRT.

Radiomics studies in esophageal cancer started relatively late, and there are still few data about applying radiomics analysis to evaluate the prognosis of esophageal cancer. Ganeshan et al. [26] first analyzed the radiomic features of CT before treatment in esophageal cancer patients and found that the radiomic features representing uniformity parameters were significantly different between stage I/II and stage III/IV disease, which were independent predictors of patients' prognosis. Subsequently, Yip et al. [14] found that the tumor heterogeneity could be represented by the change of CT radiomic features before and after neoadjuvant treatment, which was related to the prognosis and survival of patients. Larue et al. [15] also found that five radiomic features extracted from CT before chemoradiotherapy could be used to describe the tumor heterogeneity and predict the 3-year survival rate of patients after neoadjuvant chemoradiotherapy and surgery with AUCs (AUC, area under the receiver) of 0.69 in the training group and 0.61 in the validation group. All these studies suggested that radiomic features played an important role in evaluating the prognosis of esophageal cancer and could be used to predict the long-term survival of esophageal cancer patients after chemoradiotherapy, which was also supported in our study.-

Clinical TNM staging before treatment is still the most commonly used prediction system of prognosis for ESCC patients treated with chemoradiotherapy. Combination of TNM staging and other prognostic factors can predict the prognosis of patients more individually and accurately [27, 28]. Some studies have shown that the prognosis of patients who achieved CR after chemoradiotherapy was better than that of patients not CR [29, 30]. Therefore, CR after CCRT had become another important predictor for the prognosis of patients besides clinical stages. In the present study, univariate analysis showed that pre-treatment clinical T stage, N stage, clinical stage and CR after radiotherapy were the prognostic factors related to LPFS after CCRT. Moreover, Rad-score based on 17 radiomic features extracted from CT images before chemoradiotherapy was significantly related to LPFS of patients after chemoradiotherapy. Further multivariate analysis showed that Rad-score, N stage and CR after radiotherapy were independent predictors of patients' LPFS, while T stage and clinical stage had no statistical significance in this multivariate analysis model, probably because Rad-score derived from the primary tumor focus and had interactive effects with T stage and clinical stage. We developed and validated a nomogram model based on the results of multivariate analysis. C-index and calibration curve were used to evaluate the performance and prediction accuracy of the nomogram model. The C-index of the model was 0.745(95% CI 0.700–0.790) in the training cohort and 0.723(95% CI 0.654–0.791) in the validation cohort, indicating high prediction performance. The calibration curve also showed a high prediction accuracy. Therefore, we believe that this prediction model based on Rad-score can provide a more accurate tool to predict LPFS, which was a convenient and economical means.

Although a prognosis prediction model was established and validated, there are some challenges for interpretation of the results. Due to the fact that the machine and scanning parameters of CT in other centers are usually different and not standardized, the utility of the results or the radiomic features in other study was full of uncertainty. Moreover, radiomic-biology correlations have not yet to be identified in published literature and clinical experience, so there is no concrete interpretation about the features or the feature sets. On the other hand, different methodologies for feature selection and the focus on different feature sets could have led to different results. These issues have also been addressed in other studies [31].

Our study provides a good enlightenment to the coming studies to prospectively establish patient cohorts. However, there are some defects worth noting. First of all, this study is a retrospective study. Because of the long-time span of CT images used in image data acquisition, there were inevitably some problems that the image quality and scanning parameters were hard to be exactly the same, especially the development time and dosage of enhancer, so this study only collects the information of plain CT images. Secondly, as patients’ response to chemoradiotherapy could not be evaluated pathologically, clinical CR used in our study can't represent pathological CR completely truly. Fortunately, a considerable number of patients achieved CR had been confirmed by gastroscopy pathology. Being limited by the nature of a single-center retrospective study, the results may be biased to some extent, and its reliability and universality still need different centers to further carry out large sample size research verification.

## Conclusion

In a word, this study established and validated a prediction model based on radiomic features and clinical factors, which can be used to predict LPFS of patients after CCRT. As an intuitive and convenient prediction method, this model is conducive to identifying the patients with ESCC benefited more from CCRT.

## Supplementary Information

**Additional file 1.** The list of radiomics features extracted from the delineated VOIs.

## Abbreviations

ESCC: Esophageal squamous cell cancer
CCRT: Concurrent chemo-radiotherapy
CR: Complete response
LPFS: Local progress-free survival
LASSO: Least absolute shrinkage and selection operator
AUC: Area under the receiver
EC: Esophageal cancer
CT: Computed tomography
MR: Magnetic resonance
3DRT: Three-dimensional conformal radiation therapy
GTV: Gross tumor volume
GTVnd: Nodal gross tumor volume
CTV: Clinical target volume
CTVt: Tumor clinical target volume
CTVnd: Nodal clinical target volume
PTV: Planning target volume
VOI: Volume of interest
GLCM: Gray-level co-occurrence matrix
GLSZM: Gray-level size-zone matrix
GLRLM: Gray-level run-length matrix
NGTDM: Neighboring gray-tone difference matrix
GLDM: Gray-level dependence matrix

## Declarations

### Availability of data and materials

The datasets used and/or analyzed during the current study are available from the corresponding author upon reasonable request.
